# Factors influencing adherence to home-based strength and balance exercises among older adults with mild cognitive impairment and early dementia: Promoting Activity, Independence and Stability in Early Dementia (PrAISED)

**DOI:** 10.1371/journal.pone.0217387

**Published:** 2019-05-23

**Authors:** Jennie E. Hancox, Veronika van der Wardt, Kristian Pollock, Vicky Booth, Kavita Vedhara, Rowan H. Harwood

**Affiliations:** 1 Division of Primary Care, School of Medicine, University of Nottingham, Nottingham, United Kingdom; 2 Division of Rehabilitation, Ageing and Well-being, School of Medicine, University of Nottingham, Nottingham, United Kingdom; 3 School of Health Sciences, University of Nottingham, Nottingham, United Kingdom; University of Malaya, MALAYSIA

## Abstract

**Background:**

Older adults with dementia are at a high risk of losing abilities and of accidental falls. Promoting Activity, Independence and Stability in Early Dementia (PrAISED) is a 12-month person-centred exercise and activity programme which aims to increase activity and independence whilst reducing falls in people with early dementia. In this patient group, as well as many others, poor adherence to exercise interventions can undermine treatment effectiveness. We aimed to explore patterns of barriers and facilitators influencing PrAISED participants’ adherence to home-based strength and balance exercises.

**Methods:**

Participants were a subsample of 20 individuals with mild cognitive impairment or early dementia and their carer(s) taking part in the PrAISED programme. Participants (with the support of a carer where necessary) kept a daily exercise diary. Participants’ adherence were categorised based upon reported number of times a week they undertook the PrAISED strength and balance exercises over a 4 month period (<3 times a week = low adherence, 3–4 = meeting adherence expectations, >5 = exceeding adherence expectations). Semi-structured interviews were conducted in month 4 of the PrAISED programme to explore barriers and facilitators to adherence. A mixture of deductive and inductive thematic analysis was employed with themes categorised using the Theoretical Domains Framework.

**Findings:**

Participants completed on average 98 minutes of home-based strength and balance exercises per week, 3.8 sessions per week, for an average of 24 minutes per session. Five participants were categorised as exceeding adherence expectations, 7 as meeting adherence expectations, and 8 as low adherers. Analysis of interview data based on self-reported adherence revealed six interacting themes: 1) routine, 2) practical and emotional support, 3) memory support, 4) purpose, 5) past experiences of sport and exercise, and 6) belief in and experience of benefits.

**Conclusions:**

Identifiable cognitive, psychological, and practical factors influence adherence to exercise, and should be addressed in future development of interventions with this population.

## Introduction

Dementia is a clinical syndrome caused by neurodegenerative diseases and is characterised by an irreversible and progressive loss of cognitive functions (e.g., short-term memory, executive function) and associated neuropsychiatric symptoms (e.g., depression, anxiety) which can impair individuals’ ability to perform everyday activities [1]. In 2015, there were 46.8 million people worldwide living with dementia with the prevalence set to rise to 131.5 million people by 2050 [2]. The rising number of individuals living with dementia poses personal, social and economic challenges in terms of meeting increased demands and costs of care. The worldwide cost of dementia was estimated at US$ 818 billion in 2015 and projected to rise to US$ 2 trillion by 2030 [2]. Thus, dementia is a global public health priority with a pressing need for interventions aimed at preventing functional deterioration and dependency to help people live well with dementia [3].

Mild cognitive impairment (MCI) is a clinical syndrome where cognitive decline is greater than expected for the person’s age and education level, in the absence of functional decline, and can be used to identify people at risk of developing dementia [4]. Regular exercise engagement is associated with a variety of benefits for people with MCI or early dementia including improvements in activities of daily living, mobility, and mood [5–7] and a reduced risk of falls [8]. In order to obtain such benefits, older adults should engage in at least 180 minutes of strength and balance challenging exercise a week [9], with an optimal frequency of three times a week [10]. Unfortunately, many older adults with MCI/early dementia are not sufficiently active to achieve these benefits. Exercise is a subset of physical activity and defined as planned, structured and repetitive bodily movement performed to improve or maintain physical fitness [11]. A longitudinal cohort study found that physical activity levels decline in people with early dementia [12]. As a subcomponent of physical activity, it is likely that exercise participation is even lower. Thus, a key challenge with exercise interventions targeting this population is how to achieve sufficient adherence for participants to benefit.

Adherence is the degree to which a participant’s behaviour (e.g., exercise engagement) corresponds to the given agreed recommendation [13]. Reported adherence to home-based exercise interventions in people with dementia is known to vary. In a 6-month home-based, carer-supported, individually-tailored balance and strength exercise programme for people with dementia average adherence was 45% with only 22 participants (55%) completing the programme [14]. In contrast, an intervention involving one hour of home-based exercise twice a week for 12 months [8], reported high adherence with a median of 81 (out of a possible total of 104) home-based exercise sessions undertaken (78%). It is unknown what factors influence exercise adherence in this population.

Recent systematic reviews [15, 16] have identified a large number of general barriers (e.g., physical limitations, low energy levels, and burden on caregivers), facilitators (e.g., mental strategies, caregiver support and routine) and motivators (e.g., perceived benefits, enjoyment, and being with people) for exercise and physical activity engagement in people with dementia. However, previous research has not taken into account the demonstrated/actual adherence level of the participants. Thus, we do not know whether or how barriers and facilitators vary according to the degree to which a person is adherent. For example, it may be that there are particular barriers (e.g., level of cognitive impairment) that are more common among those with lower adherence, or facilitators (e.g., supportive carer) which is more common with those with higher levels of adherence. Identifying patterns of barriers and facilitators according to adherence behaviour will enable future interventions with this population to be more effectively tailored to the needs of specific individuals and designed and delivered in a way which promotes optimal motivation and adherence.

All participants were involved in undertaking home-based, strength and balance exercises as part of the Promoting Activity, Independence and Stability in Early Dementia (PrAISED) study [17]. The aim of this study was to explore patterns of barriers and facilitators influencing PrAISED participants’ adherence to the home-based strength and balance exercises.

## Method

### Design

The PrAISED programme is multi-component and consists of home-based strength and balance training [18] and functionally-orientated therapy [19] delivered by a multi-disciplinary team of physiotherapists, occupational therapists and rehabilitation support workers. All participants receiving the intervention were given an individually-tailored programme incorporating strength and balance exercises (based on the Otago programme [20]) and were encouraged to do these exercises (referred to in this study as PrAISED exercises) *at least 3 times per week* (supervised and unsupervised) but more if possible.

Data on reported adherence to the PrAISED exercises were obtained using daily exercise diaries completed by the participants and/or their carer. Semi-structured interviews were conducted to enable exploration and understanding of participants’ experiences, opinions, feelings and attitudes in relation to the factors influencing their adherence to the PrAISED exercises [21]. Ethical approval was granted by the NHS Health Research Authority (Yorkshire and The Humber—Bradford Leeds Research Ethics Committee). During the recruitment process capacity to consent was determined by researchers trained in assessing mental capacity in accordance with the Mental Capacity Act 2005 and written informed consent was gained from all participants. Findings are reported in accordance with the Consolidated Criteria for Reporting Qualitative Research guidelines [22].

### Participants

Participants were a subsample of 20 individuals (16 male, 4 female; mean age = 76.6 years, range = 68–91 years) taking part in the PrAISED feasibility trial and their carers (n = 19; 17 female, 2 male). Participants all had a diagnosis of MCI or early dementia (1 MCI, 9 Alzheimer’s disease, 4 vascular dementia, 4 mixed, 2 unknown). A detailed description of recruitment, inclusion/exclusion criteria and randomisation are provided elsewhere [17].

In the PrAISED feasibility trial, the active intervention was delivered in two ways [17]. The same content was provided in each group but with varying degrees of supervision: medium intensity and high intensity. Participants in the 3-month medium intensity programme received 14 contacts from clinicians (six occupational therapist, five physiotherapist face-to-face, plus three therapist phone calls), with 2 visits in the first week followed by weekly visits thereafter. Participants in the high intensity 12-month programme received 51 face-to-face contacts (6 occupational therapist, 5 physiotherapist, and 39 rehabilitation support worker), initially delivered twice weekly for 3 months then tapered to once a month by month 12. Participants for this study were purposively recruited to obtain a balance of views from those participants taking part in the medium intensity (n = 10) and high intensity programmes (n = 10) and a spread of high and low adherence, with participants randomly selected from within each intensity and adherence group.

### Procedure

Demographic information including (age, gender, level of cognitive impairment and self-reported physical activity) were collected at baseline. Level of cognitive impairment was measured using the standardised Mini-Mental State Examination [23]. Baseline self-reported physical activity was measured using the International Physical Activity Questionnaire (IPAQ) [24]. Interviews were conducted 4 months following the first clinician visit. At this stage of the intervention, participants in the medium intensity programme had received all of their contacts and had been encouraged to continue with the exercises without clinician supervision. Participants in the high intensity programme were receiving weekly face-to-face contact with the clinicians.

#### Exercise diaries

Participants (with the support of a carer where necessary) kept an exercise diary recording the total number of minutes of PrAISED exercises undertaken per day [17]. Participants returned the diaries to the research team monthly.

#### Interviews

Potential participants were telephoned by the lead researcher (JH) and asked if they and/or their carer would be interested in taking part in an interview about their experiences of the PrAISED programme. It was explained that participation was voluntary and that they were free to withdraw at any point during the interview. All contacted participants agreed to be interviewed and a suitable time and date arranged. All interviews were conducted face-to-face, in the participants’ home by the lead researcher, a female post-doctoral researcher trained in qualitative interviewing, and not involved in intervention delivery. The researcher talked through an information sheet and gained written informed consent prior to commencing the interview. Participants were not known personally to the interviewer. Participants and their carer(s) were given the option of either being interviewed together or individually. Most dyads (n = 15) expressed a preference for being interviewed together. Three participants and their carer(s) were interviewed separately, 1 participant had two carers present, and 2 participants did not have a carer available for interview. All interviews were audio-recorded and lasted approximately 2 hours.

A semi-structured interview guide was created based on the research teams’ expertise in exercise psychology and feedback from a Dementia Patient and Public Involvement group. Questions aimed to explore participants’ feelings and motivations related to engagement with the PrAISED exercises (e.g., How do you feel when doing the exercises? How important is it to you that you do the exercises? Are there any things that make it hard for you to do your exercises regularly?). Immediately following completion of each interview the lead author made notes about the interview environment and any observations (e.g., regarding non-verbal expressions) that may be deemed helpful for interpreting the data.

### Data analysis

#### Exercise diaries

Data on the number of minutes of PrAISED exercises undertaken per day for the four months prior to the participant being interviewed were entered into IBM SPSS Statistics for Windows, version 24 (IBM Corp., Armonk, N.Y., USA). For each month the mean was calculated for reported: number of sessions per week (total number of sessions, divided by the number of days that month, multiplied by 7), number of minutes of PrAISED exercises undertaken per week (sum of all minutes, divided by the number of days that month, multiplied by 7), and duration of sessions (total minutes per month divided by total number of times per month). A total mean and range across all 4 months was then calculated for each of the above. Participants’ adherence was categorised based on the total mean number of PrAISED exercise sessions reported per week (<3 times a week = low adherence, 3–4 = meeting adherence expectations, >5 = exceeded adherence expectations) undertaken over the 4 months.

#### Interviews

Interview data were transcribed verbatim, anonymised, imported into NVivo (Version 11, QSR, Southport, UK) and analysed using thematic analysis [25]. Thematic analysis is not bound to a particular epistemological position and was chosen as it fits with the pragmatic approach [26] adopted within this study. All interviews were coded by the first author (JH). She familiarised herself with the data via ‘repeated reading’ and noting initial meanings and patterns. The data were coded inductively for perceived barriers and facilitators to engaging in the PrAISED exercises. An initial coding frame was developed, using the principle of constant comparison, to include data-driven themes and patterns. VvDW and KP analysed a sub-set of the data; 2 transcripts were coded by all three researchers and 4 transcripts double coded (30% of interviews). Following discussions and collaborative reflections with VvDW and KP, themes were identified, critically reviewed and adapted until a consensus was reached. All authors agreed that that point of data saturation had been reached with no significant new themes emerging.

Following inductive thematic analysis, the generated themes were considered deductively in relation to the Theoretical Domains Framework (TDF) version 2 [27, 28]. The TDF is a theoretical framework synthesising key theoretical constructs of motivation and behaviour change into 14 domains (see Cane et al. [27] for descriptions of each domain). The TDF has been applied systematically to assess barriers and facilitators in relation to a range of healthcare behaviours [28, 29]. The framework analysis method [30] was used to facilitate comparing and contrasting of data based on adherence group. A spreadsheet was created to chart the data (with themes in relation to the TDF along the vertical axis and participants along the horizontal axis). The charted data were then analysed separately for themes within each adherence group (exceeded expectations, met expectations, or low self-reported adherence). A clear audit trail, documenting analytic decisions was created and maintained to maximise transparency and ensure credibility and quality.

## Results

In the first four months of taking part in the PrAISED programme, on average participants completed 98 minutes of PrAISED exercises per week (range = 22–464), 3.8 sessions per week (range = 0.9–7.0), for 24 minutes per session (range = 14.5–66). Five participants’ adherence exceeded expectations (4 male, 1 female; mean age = 75.40, range = 70–80 years; mean sMMSE score = 25.20, range 21–27; 3 medium intensity, 2 high intensity programme). Seven participants met adherence expectations (all male, mean age = 79.00, range = 69–91 years; mean sMMSE score = 26.43, range = 24–30; 1 medium intensity, 6 high intensity programme), and 8 participants had low adherence (5 male, 3 female; Mage = 75.25, range = 68–86 years; mean sMMSE score = 23.87, range = 20–29; 6 medium intensity, 2 high intensity programme) (Table 1).

**Table 1:** Sample characteristics and adherence grouped based on number of PrAISED exercise sessions per week.

Participant														Carer
Case	Pseudonym	Gender	Age (yrs)	sMMSE ^a^	Intervention group	Baseline IPAQ ^b^	Number of times PrAISED exercises per week		Total mins of PrAISED exercises per week		Duration (mins) of PrAISED exercises per session		Adherence Group ^c^	Gender & relationship
							Mean (SD)	Range	Mean (SD)	Range	Mean (SD)	Range		
1	Mr Davis	M	79	27	Medium intensity	1344.0	7.0 (0.0)	7.0–7.0	464.0 (44.4)	70–501	66.3 (6.3)	57–72	Exceeded	F, daughter
2	Mr Clarke	M	73	27	Medium intensity	9723.0	7.0 (0.0)	7.0–7.0	209.2 (38.1)	169–210	29.9 (5.4)	24–35	Exceeded	F, spouse
3	Mrs Patterson	F	80	26	High intensity	33.0	7.0 (0.0)	7.0–7.0	157.5 (35.0)	140–210	22.5 (5.0)	20–30	Exceeded	F, daughter
4	Mr Johnson	M	70	25	Medium intensity	3807.0	6.1 (0.6)	5.3–6.8	118.5 (17.3)	95–135	19.5 (0.95)	18–20	Exceeded	F, spouse
5	Mr Evans	M	75	21	High intensity	198.0	6.9 (.25)	6.5–7.0	107.3 (15.5)	95–130	15.7 (2.9)	14–20	Exceeded	F, spouse
6	Mr Jenkins	M	80	25	High intensity	2712.0	3.6 (1.1)	2.5–5.2	132.3 (38.3)	83–176	36.8 (3.8)	33–41	Met	F, spouse
7	Mr Hughes	M	80	30	High intensity	594.0	3.5 (2.0)	0.9–5.4	89.3 (38.1)	34–121	28.7 (8.2)	21–38	Met	F, spouse
8	Mr Edwards	M	69	28	High intensity	406.5	3.4 (0.8)	2.8–4.5	77.7 (11.3)	67–90	23.2 (4.7)	20–37	Met	F, spouse
9	Mr Williams	M	69	25	High intensity	1485.0	4.6 (2.0)	1.8–6.5	76.5 (26.2)	40–101	17.6 (2.9)	16–22	Met	F, spouse
10	Mr Thompson	M	75	24	Medium intensity	2071.5	3.2 (0.5)	2.7–3.7	67.6 (12.3)	53–79	21.7 (4.9)	17–28	Met	F, spouse
11	Mr Lewis	M	91	29	High intensity	958.5	4.3 (0.7)	3.5–5.0	66.0 (9.9)	56–78	15.4 (0.6)	15–16	Met	Unavailable
12	Mr Roberts	M	89	24	High intensity	376.0	3.6 (2.8)	1.6–5.6	36.2 (28.1)	16–56	10.0 (0.0)	10–10	Met	F, spouse
13	Mr Taylor	M	75	29	Medium intensity	792.0	2.4 (1.4)	1.1–4.0	82.7 (65.5)	34–177	33.0 (10.5)	24–45	Low	F, spouse
14	Mr Harris	M	68	20	Medium intensity	930.0	2.9 (1.1)	2.0–4.3	78.3 (20.4)	54–99	27.7 (4.5)	22–23	Low	F, spouse & F, daughter
15	Mr Wilson	M	86	29	Medium intensity	1189.5	2.0 (0.6)	1.4–2.7	46.9 (12.8)	32–63	23.4 (0.7)	23–24	Low	F, spouse
16	Mrs Davies	F	75	21	Medium intensity	148.5	2.3 (0.7)	1.4–2.9	38.9 (13.5)	20–52	18.6 (9.1)	10–31	Low	M, spouse
17	Mrs Smith	F	71	22	Medium intensity	536	2.1 (0.8)	1.6–3.4	28.9 (12.3)	18–46	13.4 (2.4)	10–16	Low	M, spouse
18	Mr Wood	M	80	21	High intensity	2712.0	0.9 (0.3)	0.7–1.4	26.5 (8.3)	20–38	29.4 (1.8)	28–32	Low	F, spouse
19	Mrs Brown	F	77	25	High intensity	438.0	1.6 (0.5)	0.9–2.0	26.4 (6.1)	18–33	17.1 (2.4)	14–20	Low	Unavailable
20	Mr Harrison	M	70	24	Medium intensity	1071.0	1.6 (0.7)	0.7–2.3	22.3 (10.8)	7–32	13.5 (2.4)	10–15	Low	F, partner

^**a**^ sMMSE = level of cognitive impairment (score out of 30) measured using the standardised Mini-Mental State Examination [23]

^**b**^ IPAQ = International Physical Activity Questionnaire [24]. Results are reported as total MET-minutes/week. Physical activity classification criteria: ≥ 3000 MET-minutes/week = high, ≥ 600 MET-minutes/week = moderate, < 600 MET-minutes/week = low (see www.ipaq.ki.se for further details).

^**c**^ Adherence group: low = <3 times a week, met = 3–4, exceeded = >5 times per week)

Qualitative findings revealed a variety of barriers and facilitators (covering all 14 TDF domains [27]) reported by participants and their carers to adherence to the PrAISED exercises (Table 2). Analysis of themes based on participant self-reported adherence identified six interacting themes: routine, practical and emotional support, memory supports, past experiences of sport and exercise, purpose and beliefs in and experience of benefits.

**Table 2:** Barriers and facilitators perceived to influence adherence to the PrAISED exercises mapped using the Theoretical Domains Framework.

TDF	Facilitators	Barriers
1. Skills	• Tailoring of exercises to individual’s ability level • Practice	• Reduced mobility or pain due to illness or injury • Visual impairment
2. Knowledge	• Demonstration of exercises by clinician • Instruction on how to perform the exercises • Information about health consequences/rationale for specific exercises • Pictures of exercises	• Being unclear on rationale for specific exercises
3. Memory, attention and decision processes	• Memory supports - Prompts/cues from clinicians, carer and/or pictures of exercises as a reminder as to what exercises to do and how to do them - Prompts/cues from clinicians, carer and calendar as to when do to the exercises	• Memory problems resulting in inability to remember when to do the exercises, how to do the exercises and/or why to do the exercises.
4. Behavioural regulation	• Routine (habit-formation) - routine of when, where and how the exercises are performed - regular behavioural practice until exercises became automatic • Self-monitoring when and how long they do the exercises acted as a prompt and reflection tool	• No routine to when or how the exercises are performed • Dislike of routines and feeling controlled
5. Environmental context and resources	• Home-based–no need to travel • Exercises do not take long to do	• Other activities/events (e.g., hobbies, birthdays, holidays, looking after grandchildren)
6. Social influences	• Practical and emotional support - From clinician and carer - Carer belief that exercise is beneficial - External social support—group exercise class	• Feeling pressured due to clinician expectations • Loss of autonomy/feeling controlled • Carer burden
7. Social role and identity	• Positive past experiences of sport and exercise involvement • Active disposition	• Negative experiences/dislike of prescriptive exercises
8. Beliefs about capabilities	Feeling competent performing the exercises Feeling optimally challenged	• Belief that they are fit enough/are not a falls risk • Belief that they get exercise in other ways
9. Optimism	• Optimistic that exercises will be of benefit in the long-term	
10. Beliefs about consequences	• Believing in and experiencing benefits - Belief that doing the exercises will be beneficial (physically and/or mentally) - Seeing improvements strengthened belief in benefits of the exercises • No perceived harm or risk	• Not sure if doing the exercises will be beneficial (physically and/or mentally) • Not seeing improvements decreased perception of benefit • Perceived harm or risk
11. Intentions	• Strong intention to continue with exercises	• Lacking intention to continue with exercises
12. Goals	• Purpose—personalised, meaningful goals focused on facilitation (e.g., helping participants to continue to do activities they enjoy doing). • Carer involvement in goal setting	• Not seeing the point/purpose of doing the exercises
13. Reinforcement	• Constructive feedback from clinician on technique • Verbal encouragement/praise from clinician	
14. Emotion	• Enjoyment • Feeling of achievement • Guilt–not wanting to let clinician or carer down	• Apathy–lack of energy and interest • Depression • Fear of falling • Frustration at deterioration in ability

Participants exceeding adherence expectations reported key facilitators to be: development of a daily exercise routine, strong practical and emotional support from the clinicians and their carer, use of memory supports, positive past experience of sport and exercise involvement, purpose for doing the exercises, and believing in and experiencing benefits from doing the PrAISED exercises. Participants who met expectations or who had low adherence reported lower levels or a lack of one or more of these facilitators.

Although factors (e.g., depression) within other theoretical domains (e.g., emotion) were reported as barriers or facilitators for certain individuals, no pattern was identified based on adherence level. For example, individuals from all adherence groups reported depression as a barrier but with effective practical and emotional support and development of a routine participants with depression in the exceeding adherence expectations group were able to overcome this.

### Interactions

**Fig 1:**
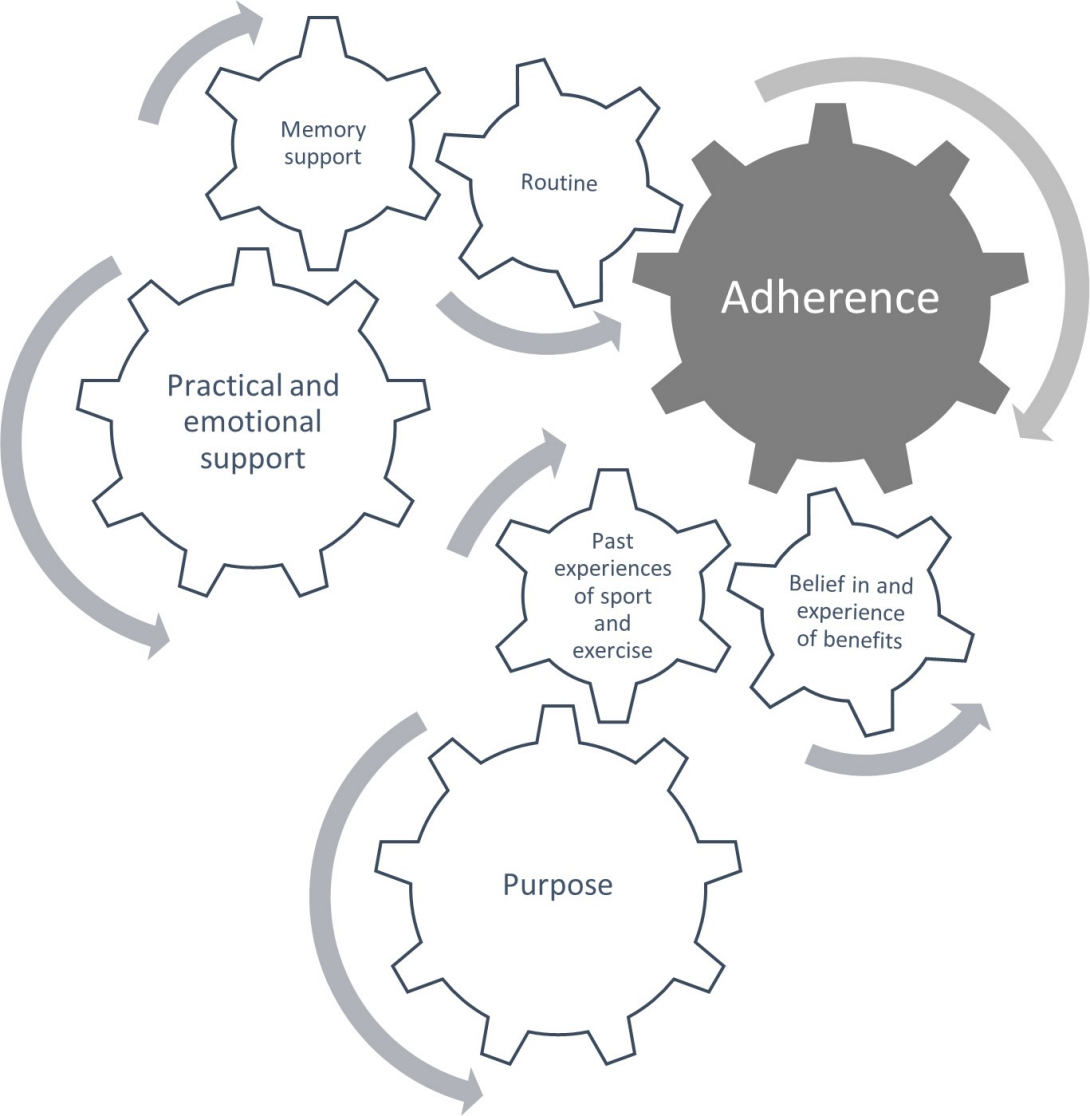
Illustration of interactions between themes.

Fig 1 illustrates the interactions between themes. The themes of ‘routine’, ‘memory supports’ and ‘practical and emotional support’ were all closely interlinked. The use of memory supports (which included practical support from clinicians and carers in the form of prompts) helped performing the exercises to become a habitual routine which, in turn, acted as a memory support for adhering to the exercise programme.

The themes ‘past experience of sport and exercise’, ‘purpose and beliefs in and experience of benefits’ were closely linked, with high adherence participants’ purpose being to continue doing the activities that they enjoy doing (e.g., a past or current sport or physical activity), which was described as a key part of their identity. Their beliefs about the benefits of exercises (influenced by their past experiences of sport and exercise) were also a key motivator for doing the exercises which was reinforced through seeing benefits from doing the PrAISED exercises.

Participants with low adherence either 1) lacked purpose (did not see the point in doing the PrAISED exercises believing themselves to not be at a falls risk and/or disliked structured exercise and feeling that they got enough exercise from other activities) and/or 2) were below a threshold of cognitive capability and did not have adequate practical and emotional support (i.e., a carer who is willing and able to assist participants to perform the exercises unsupervised between clinician visits and develop an exercise routine).

### Theme 1. Routine

#### Daily exercise routine

All participants who exceeded adherence expectations reported having got into a routine of doing exercises on a daily basis but were generally flexible with what time of day they did the exercises.

*I don’t even consider what time it is…if we have to go out in the morning*, *then I do them [the exercises] in the afternoon*. [Mr Clarke]

Two of the participants who exceeded adherence expectations explained that the exercises had become a way of life now. One described how he had got into a routine of doing the PrAISED exercises whilst his wife prepares their dinner.

*But*, *now it’s second nature really*, *I suppose*. *And to do exercises at a certain time of day*, *in the evening*, *usually do it when [carer] is getting the evening meal… Been doing them for so long that it just becomes automatic*, *and you think*, *this is what I do while the evening meal is being prepared*. [Mr Johnson]

The way in which the participants who exceeded adherence expectations completed the exercises had also become habitual. Participants tended to have a particular place where they did certain exercises. They reported doing the exercises in the same order each time using pictures of the exercises provided by the physiotherapist as prompts as to how to do the exercises. Despite their memory impairments, all participants who exceeded adherence expectations reported that after a few months they no longer needed to use the pictures of the exercises on a regular basis and only used them every now and again as a reminder.

*I used to turn over the pages and then I got used to what I have to do*, *which is good*. *I’m more confident than I used to be at doing the exercises… I can do them without the pieces of paper now*. *I can remember them* …*in my own brain*, *which is a good thing*. [Mrs Patterson]

#### Flexible exercise routine

Five of the seven participants who met adherence expectations felt that after four months of the programme, doing the exercises regularly had become a routine, but a flexible one. They liked the flexibility to choose which days of the week they did the exercises to fit in with their other activities. For example, Mr Williams tended to do the exercises on Mondays, Wednesdays and Fridays, as he does other activities he enjoys on Tuesdays and Thursdays (which included walking the dog, Men’s Shed–a community group focused on making and mending things, and attending a group exercise class).

Two participants who met adherence expectations felt that they were still in the process of the exercises becoming routine with their engagement based on how they were feeling that day.

*Well*, *say*, *it’s becoming a routine* … *still building up to that process*. *When you do get up in the morning it may not necessarily be a chosen day but*, *getting up and doing it*, *it’s a part of a routine that*, *in that week*, *you’ve got to do some exercises*. *The flexibility is handy*, *because*, *if you are feeling a bit*, *and I do wake up feeling a little bit down*, *tired…I can spread it through the week*. [Mr Edwards]

All seven participants who met adherence expectations described using the pictures of the PrAISED exercises to prompt them how to do the exercises, with four participants reporting to have learned the exercises and no longer needing to use the pictures on a regular basis. Participants who met adherence expectations tended to stick less to a set order when doing the exercises compared to those exceeding adherence expectations. For example, one participant described how he breaks the exercises up into smaller chunks to fit in between his other activities.

*Sometimes*, *I do the full programme*, *and then*, *another time*, *I do the arms*, *and the other time*, *I do the legs*. [Mr Jenkins]

#### No exercise routine

All eight participants with low adherence reported having no routine in relation to doing the PrAISED exercises.

*I’ve got no set time or whatever*, *to do things*. *If I want to do them*, *I do them*. *If I don’t*, *I’ll not*. *End of story*. [Mr Taylor]

Low adherence participants reported not liking routines because of not wanting to feel controlled.

*Mr Smith [carer]*: *It’s been suggested that we could try a routine, whether we do it [the exercises] before breakfast or…*

*Mrs Smith [participant]*: *I don’t like routines. They irritate me. And…*

*Mr Smith [carer]*: *Any form of routine, I think, she means. You don’t like being …*

*Mrs Smith [participant]*: *Controlled from afar.*

### Theme 2. Practical and emotional support

#### Strong practical and emotional

Social influences were a key motivating factor for all participants, however, those with higher levels of adherence tended to report a greater sense of practical and emotional support from their clinicians and carer. For one of the participants exceeding adherence expectations, supervision and encouragement from both the clinicians and their carer was vital to their adherence as the participant had a high fear of falling and did not feel comfortable doing the exercises alone: “I try and do them when somebody’s about, so I don’t fall.” [Mrs Patterson]

Regular visits from the clinicians were highly valued by both the participants and their carers. It was felt that participants were more responsive to external support from the clinicians than they would be if it had come only from family members. Particularly for participants with greater levels of cognitive impairment, clinician support was vital.

*“[Mrs Smith] responded better to the physio in the way of… she went through the exercises with her*, *every time she visited really*. *Which was a success for me because sometimes you have to make yourself do these things*. *I have to be aware to remind Mrs Smith and put her through this exercise programme*. *She responds easier when there’s someone [a clinician] here to do it*. [Mr Smith, carer]

Participants reported finding the development of a rapport with the clinician a key facilitator. Participants reported feeling motivated when the clinicians provided good two-way communication, clear instructions, a rationale for the exercises, and tailored the exercises to their interests and needs. The relationship between the clinician and participant was viewed as important to engagement with valued features of the relationship being: a shared sense of humour, similar interests, and a sense of trust and understanding. For example, one participant who met adherence expectations explained:

*Well*, *because she seems to understand my mental attitude*, *she understands my physical limitations*, *she understands what I can do*, *how to give me positive help…I think the*, *the physio knows what she’s talking about*. *I’ve a lot of faith in her*. [Mr Hughes]

Participants with high adherence had carers who reported believing exercise to be beneficial and who were supportive of the participants’ involvement in the programme.

*I’m am a big believer that if you are physically healthier*, *then your mental health improves*. [Mrs Johnson, carer]

*We’ve always supported each other in what effort we do anyway*, *so*, *it’s just one of those things*. [Mrs Clarke, carer]

Carers of participants exceeding adherence expectations greatly valued being involved and receiving emotional support provided by the clinicians.

*They [the clinicians] were always sure that you knew what they’d done and that you understood what they were doing…Even if it’s just an ear sometimes*, *maybe that wasn’t what they were here for*, *but they were just very supportive*. [Mrs Johnson, carer]

The support from the clinicians enabled the carers to support participants to do the exercises.

#### Lack of practical and emotional support

Six of the eight participants with low adherence were in the medium intensity group so received less support from clinicians which put more reliance on carers to support participants to do the exercises. For some participants carer prompts to do the exercises led to the participants being less likely to want to do the exercises (feeling a loss of autonomy).

*I’m not sure that it [prompts from his partner to do the exercises] motivates me*. *Because I’m*, *I’m a man*, *I guess*, *and if I’m told to do something*, *the natural resistance is to say ‘on your bike'*. [Mr Harrison]

Participants with low adherence tended to have carers who found set exercises themselves boring (e.g., Mr Taylor, carer–‘…because there’s nothing more boring than exercising’), did not see the purpose of doing specific PrAISED exercises, had a preference for functional activities (e.g., walking, gardening) and/or who were experiencing a sense of burden supporting their spouse to do the exercises. For example, Mr Smith (carer) described how he lacked the motivation himself and believed that his wife was active enough already and did not currently have any physical problems.

*I think that*, *as far as I’m concerned*, *I mean*, *I think [the participant] would do them if I had the discipline to make sure she did them… And I think*, *at the same time*, *I know they are important but as*, *[the participant] being quite active anyway*, *it’s like an added thing*, *isn’t it*, *to do exercises*? [Mr Smith, carer]

Mrs Taylor (carer), who prior to the programme was struggling to deal with her husband’s diagnosis of depression, found it very stressful trying to motivate her husband to do the exercises unsupervised between visits:

*The only negative thing was the pressure put on us in the first place [to do the exercises]*. *Erm*, *which was probably more me than [the participant] to be honest*. *Because I was trying to do what*, *what I was told to do*, *and I was putting pressure on [the participant] and then*, *he dug his heels in*. *And then I got cross*, *inside*, *I didn’t shout at him*. *Because*, *there’s no point*, *but just wound me up*. *But that was*, *that was the biggest negative*. [Mrs Taylor, carer]

### Theme 3. Memory supports

#### Use of memory supports

Despite experiencing memory problems, participants with higher levels of adherence had found memory support strategies that worked for them. Remembering to do the exercises was facilitated by the development of a daily routine, and prompts from clinician visits and carers. To help with remembering how to do the exercises, participants used the pictures of the exercises as prompts and performed the exercises in the same way each time which led to the exercises becoming more automatic. Mr Evans (sMMSE– 21) reported particular difficulty with remembering how to do the exercises. However, he was successfully enabled to continue via strong carer involvement and support and the clinicians taking time to understand his needs. They adapted his programme so that exercises were progressed at a rate which the participant felt comfortable with. Rather than progressing the difficulty of the exercises or adding in new ones the clinicians encouraged the participant to increase the number of repetitions so that the exercise became automatic before progressing.

*There was a problem with [the participant] trying to get*, *to do exercises with cognitive building*, *it wasn’t happening*, *so they want him to do more of the exercise he’s doing now…more intense…They’re going to adapt the programme to your needs*. [Mrs Evans, carer]

#### Lack of memory supports

Three participants in the low adherence group with higher levels of cognitive impairment reported problems with both remembering to do the exercises and remembering how to do the exercises. They were less likely to make use of the pictures of the exercises themselves and tended to rely more on the clinician or their carer to remind them of how to do the exercises.

*What she’s trying to say*, *we have to remind her how to do them*. *She couldn’t do them on her own without me showing her how to do them*. *Then she’ll do it*. *And that’s just the short term memory*. [Mr Smith, carer]

All three of these participants were in the moderate intensity programme so had received fewer clinician visits and their carers all reported high levels of burden, finding it difficult to support their spouse to do the PrAISED exercises in between clinician visits.

### Theme 4. Past experiences of sport and exercise

#### Positive experiences of sport and exercise

All five participants in the exceeding adherence expectations group and most (n = 6) of the met adherence expectations group had a strong identity with being active and/or a history of positive experiences of sport and exercise involvement and this was a key facilitator to continuing with the exercises. For example, Mr Thompson described how the exercises reminded him of positive past times playing table tennis and football:

*I used to play table tennis and football in the league*, *I was semi-professional with them*. *That’s why the programme with exercises I like doing*. *Exercise is something I’ve been doing since I was sort of*, *from school*, *onwards… Used to go training twice a week*. *And then*, *continued right up until I were no longer fit enough*. *Yeah*. *So*, *it’s … when I do the exercises at home*, *it reminds me of when I was playing football in the league*. [Mr Thompson]

#### Negative experiences of prescriptive exercises

The three participants with the lowest adherence identified as having always been active but found prescriptive exercises boring, preferring to get their exercise from activities they find enjoyable (such as, walking, bell-ringing, yoga).

*Mr Harrison*: *I’ve always been pretty active. I mean, I used to go to the gym regularly, and that becomes a bore. So things like having to do these daily exercises that are set out for me, can’t be bothered quite frankly…*

*Interviewer*: *So, you’re saying you like being active but that you find exercise a bore. Is there a difference in your mind between being active and doing exercise?*

*Mr Harrison*: *Well*, *yes*, *because exercise is prescriptive… But we do lots of other things as well*, *you see*. *We’re not*, *we don’t sit in chairs… I mean*, *we walk quite a bit*, *I do a lot of [church bell] ringing which involves meeting people and climbing stairs and physical exercise*. *See I do about two or three times a week*. [Mr Harrison]

### Theme 5. Purpose

#### Meaningful purpose

Understanding the rationale for, and having a clear and meaningful purpose in relation to, doing the exercises was important. All participants with high levels of adherence reported specific reasons/purpose for doing the exercises which tended to focus on: prevention (e.g., maintain physical and psychological health), therapy to fix a problem (e.g., improve balance to reduce falls), and/or facilitation (e.g., help participants to continue to do activities they enjoy doing, such as, walking). Walking was a key motivating factor for all 5 participants exceeding adherence expectations. Mr Clarke felt that by doing the exercises his balance was improved which meant that he would be able to continue walking his dog independently, something he enjoyed doing. For Mrs Patterson doing the exercises was important to enable her to continue walking so that she can get to activities she enjoys, for example, bridge club. The number of minutes of activity self-reported by Mr Davis was particularly high. The interview revealed that in addition to the PrAISED exercises the participant had also been recording his daily walks as part of the PrAISED exercises. Mr Davis’ motivation for doing the PrAISED exercise was to enable him to continue with valued normal activities such as going out on errands that provided the opportunity for social engagement.

#### Lack of purpose

Low adherence participants who reported reasons for doing the exercises mentioned maintenance (Mr Wilson: *‘Well*, *I just take them as part and parcel of*, *you know*, *if you feel good within your body you will probably feel good in your mind’*) and prevention (Mrs Harris, carer: *‘If he thinks it’s going to not make his dementia get any worse*, *then he will do them’*). The majority of participants in the low adherence group lacked a clear purpose for doing the exercises. These participants did not see the point in doing the PrAISED exercises, believing themselves to not be at risk of falls and/or to be getting enough exercise from their other activities. For example, Mr Wood and his wife did not believe him to be a falls risk and felt that he got enough exercise from his daily activities.

*Some of them [the exercises] are to help [the participant’s] balance because*, *apparently*, *there’s a tendency to fall*, *isn’t there*? *But he’s had nothing like that*. *He’s had no symptoms of falling or dizziness or anything*. [Mrs Wood, carer]

### Theme 6. Belief in and experience of benefits

#### Strong belief in and experience of benefits

All participants who met or exceeded adherence expectations reported a strong belief in the benefits of doing exercises to be a key facilitator.

*Well*, *it’s important that I do them*, *because*, *they’re for my benefit and I’m going to prolong whatever I can as long as I can*. [Mr Thompson]

This belief was reinforced during the programme by participants experiencing physical (e.g., increased mobility, improved balance and reduced pain) and psychological (e.g., improved mood, confidence and quality of life and reduced stress and anxiety) benefits from doing the exercises.

*But*, *it’s proved*, *by doing this*, *this programme that the exercise does help you*, *and I’m sure*, *if we’d not done anything*, *I wouldn’t have been so agile on my feet as I am*, *I’d have been struggling along… And when you see that it*, *or when you know that it is beneficial to you*, *you think*, *well*, *I’m carrying on with this*, *I want to do this*. [Mr Johnson]

#### Belief in benefits not enough

Low adherence participants tended to express that exercise in general should be beneficial but did not appear entirely convinced. It became apparent that although such participants mentioned doing the exercises because of the assumed benefits, they tended to be unclear as to what the specific benefits were. Furthermore, participants with lower adherence reported not experiencing any benefits (potentially as a result of not doing the exercises regularly enough), which led to them (and their carers) feeling less motivated to adhere and continue with the exercise programme.

*I’m not very motivated to do them…*.*actually*, *I don’t see*, *in my own mind*, *how that’s [the exercises] going to improve my memory… I’m not sure that I can*, *personally*, *can see any benefit of being involved* … *Perhaps if I could see a tangible benefit*, *that would encourage me*. [Mr Harrison]

## Discussion

This paper analysed barriers and facilitators to adherence to home-based strength and balance training among older adults with mild cognitive impairment and early dementia. In line with previous research [15, 16] the study revealed a range of barriers and facilitators to exercise adherence, spanning all 14 TDF domains. This study builds on previous research [15, 16] through an examination of how barriers and facilitators varied according to levels of adherence.

On average the subsample of PrAISED participants undertook 98 minutes of strength and balance challenging exercises per week. This is considerably less than the recommended level of 180 minutes per week [9]. However, participants may have been engaging in strength and balance challenging exercises as part of their everyday activities (e.g., gardening). These were not recorded in this study but in a clinical setting may be useful for engagement and long-term adherence. Analysis of qualitative data helped us to understand the reasons underlying the variation in adherence. Based on self-reported adherence, six interacting themes each from different TDF domains were identified: memory support (*memory*, *attention and decision processes*), routine (*behavioural regulation*), practical and emotional support (*social influences*), purpose (*goals*), past experiences of sport and exercise (*social role and identity*), and belief in and experience of benefits (*beliefs about consequences*).

The findings suggest that a programme of set strength and balance exercises are unlikely to be performed by individuals below a threshold of cognitive capacity who do not have adequate practical and emotional support (i.e., a carer who is able and willing to motivate and assist participants in developing an exercise routine). Furthermore, participants who lack purpose for doing the exercises are also unlikely to adhere (i.e., do not see the point in doing the exercises, believe themselves to not be at a falls risk and/or dislike structured exercise and feel that they get enough exercise from their other activities). Previous research has reported similar findings with a dislike of structured exercise, caregiver health and availability issues, and memory loss to be reasons for lower adherence in exercise programmes with community-dwelling older adults with dementia [31].

This study found practical and emotional support from clinicians and carers to be key to adherence. It is widely assumed that presence and support of carers is important in promoting adherence [32, 33]. However, this research suggests it is crucial to consider the quality of the support provided and issues such as carer burden and the relationship dynamics between the participant and carer. To improve participant adherence future research interventions could incorporate greater emotional support for carers and information on what carers could say and do to most effectively motivate participants to exercise. Alternatively, the number and/or frequency of visits could be tailored to the individual dyad's needs, with an increased number of clinician supervised sessions for those with low cognitive capacity and high carer burden or no or little carer support. Consideration would need to be given, however, to the feasibility of a tailored, intensive support approach. A randomised controlled trial (PrAISED2) is currently being conducted to explore the extent to which such an intervention with tailored frequency of visits is feasible and economically viable [34].

Results revealed the creation of an exercise routine (when, where and how the exercises are performed becoming habitual) to be a key facilitator to adherence to the PrAISED exercises. The creation of physical activity habits has been found to aid maintenance of physical activity behaviours in a variety of populations [35]. A small body of research has noted the importance of routines for physical activity engagement in participants with dementia [15, 36, 37]. This study suggests that, despite memory problems, with the appropriate carer support, it is possible for those with MCI or early dementia to create a habitual exercise routine within a 4-month intervention. A daily exercise routine was critical for high adherence. However, flexibility to adapt the exercises to fit with their routine and other activities was viewed as important. Thus, future individual home-based exercise interventions focused on developing exercise habits should take into account participants current commitments and preferences for routine. Development of an exercise routine was an obstacle for all participants with low adherence. In order to address this key barrier to adherence, future research could explore further individuals’ negative beliefs around structured exercise routines and whether it is possible for these beliefs to be reframed through therapist support.

Another key motivator to adherence to the exercises was having a purpose. As part of the PrAISED programme, clinicians had been encouraged to explain the rationale and benefits for doing the set strength and balance exercises, conduct joint goal-setting and to, if possible, link the exercises to an activity which the participant finds enjoyable and/or meaningful (e.g., show how doing the exercises will help participants to continue to do activities they enjoy doing, such as, walking). The findings suggest that this approach worked for participants with high adherence (with all reporting having a purpose to be a key motivating factor). However, it appears that for some individuals identifying a meaningful purpose for the set strength and balance exercises was challenging. For such individuals, other types of exercise, for example exercises which are integrated into lifestyle activities [38], group exercise classes [39], or music movement therapy [40] may be more appropriate.

A novel finding was that some people met and/or exceeded adherence expectations regardless of the level of supervision (moderate or high intensity) they received. If we can identify those individuals who are likely to adhere with lower levels of supervision then resources can be diverted to those who are the most in need of the support. The findings from this study could be used to aid the development of an adherence risk screening tool which could identify those individuals who may require greater levels of support. The intervention could then be more effectively tailored to the needs of those specific ‘high risk’ individuals. For example, individuals’ below a threshold of cognitive capacity, who have a high fear of falling and/or who do not have appropriate carer support could be given greater clinician contact time and increased levels of carer emotional support. For individuals who have a strong dislike of structured exercise, rather than being given set strength and balance exercises, they could instead be encouraged to identify strength and/or balance challenging physical activities which are preferable and meaningful to them and then be supported by clinicians to engage in and maintain these physical activities. Qualitative research utilising a realist approach to explore what works, for whom, in what circumstances and why and/or quantitative research using clustering to identify characteristics of adherent participants, would provide further evidence to support the development of an adherence risk screening and tailoring tool.

### Strengths and limitations

Exercise diary and interview reports from participants and carers may have been subject to response bias. To our knowledge, this is the first study with older adults with MCI and early dementia to explore patterns of barriers and facilitators reported by participants based on different levels of adherence to completing home-based, strength and balance exercises. The use of the TDF for structuring the analytical process enabled a comprehensive and theory-derived process for identifying determinants of adherence [30]. Although individuals with MCI and early dementia taking part in a home-based exercise programme may experience a range of barriers and facilitators, this study identified six TDF domains which are particularly relevant to participant adherence. Future research, designing home-based exercise interventions for this population could use this information on key determinants of adherence to identify appropriate intervention methods (e.g., behaviour change techniques such as habit-formation and social support).

## Conclusion

This study advances our understanding of the barriers and facilitators to adherence to home-based strength and balance exercises in older adults with early dementia. Findings suggest that individuals who develop an exercise routine, have strong practical and emotional support from clinicians and carers, use of memory supports, have positive past experience of sport and exercise involvement, have a purpose for doing the exercises, and believe in and experience benefits are likely to adhere to a programme of strength and balance exercises. However, individuals below a threshold of cognitive capacity who do not have adequate practical and emotional support and/or do not identify with a meaningful purpose for doing the exercises are unlikely to adhere. The findings can be used to refine current interventions with the target population or be considered in the development of new interventions, so that home-based exercise interventions with older adults with MCI and early dementia more effectively target the key determinants of adherence and are designed and delivered in a way which promotes optimal motivation and adherence.

## Supporting information

S1 File PrAISED interview topic guide. (PDF)
