# Supplementary material for: Factors influencing adherence to home-based strength and balance exercises among older adults with mild cognitive impairment and early dementia: Promoting Activity, Independence and Stability in Early Dementia (PrAISED)
Source: PLoS One. 2019 May 23;14(5):e0217387. doi: 10.1371/journal.pone.0217387 (PMC6532965; doi:10.1371/journal.pone.0217387)
Supplement: S1 File — (PDF) [file pone.0217387.s001.pdf]

PrAISED interview topic guide

Please note this interview topic guide was designed to explore the individuals' and their carers' overall experiences of participating in the PrAISED programme. The current paper reports only on participant responses to specific sections relating to participants' experiences and support for doing the physiotherapy exercises.

Introduction

- *The aim of the interview is to find out about your experiences of taking part in the PrAISED programme.*
- *I am interested in what you thought of the programme and how you found taking part: there are no right or wrong answers, we are simply interested in what you think*
- *If you are willing, we would like to make a recording of our discussion, so that we have an accurate record of what was said.*
- *Everything you tell us is confidential, and no one except members of the PrAISED research team will know what you said. If we use something that you have said in a report we will do so anonymously – your name will not be used.*
- *You don't have to answer any questions or discuss any topics you don't want to.*
- *Do you have any questions about the interview before we begin?*
    - *Completion of consent to audio recording and to take part in the interview*

NB - the following questions are suggestions and prompts – some answers may be anticipated earlier in the discussion and others turn out to be not relevant. The interviewee may also raise additional topics and issues which they feel are particularly relevant and these should be followed up in the discussion.

General prompts and follow ups, e.g.: Can you say a bit more? How did you feel about that? Is there anything else you feel is important about this? Why do you think that is? Is there anything else you can think of about xxxx?

Note. Most questions are aimed at both the participant and carer. Where possible ask participant first, then the carer for their views. Questions starting with C: are specifically aimed at the carer.

Section 1. Recruitment/uptake

1. To start with could you tell me a bit about how you got involved in the PrAISED programme?
2. What was it that interested you about the programme?
3. Why did you want to get involved?
    - What would you say were your reasons for taking part?
    - Did you want to take part, or did you feel under pressure to do so? (from whom)
4. What were your expectations going into the programme? What did you think it would involve?
5. What did you hope to achieve from your participation in the programme?
6. Did you have any concerns about the programme? What? Why?

Section 2. Experiences of the project

1. Can you tell me a bit about your experience of being involved in the PrAISED programme? How you have actually found it so far?
2. To what extent do you think your expectations of the programme were met?
3. What do you like about the programme?
4. Have you gained any benefits from being involved in the project?
   - Have you noticed any changes to your physical or mental health since starting the programme (such as pain, energy levels, appetite, anxiety, depression, blood glucose levels if diabetic and monitoring, BP if monitoring). If yes, why do you think the changes have occurred?
5. Have you changed any health behaviours since starting the programme (diet, drinking, smoking…).
   - If yes, how and why?
6. Have you discussed with the therapists any non-exercise related problems?
   - If so, how have they responded/what have they done?
7. What don't you like about the programme?
8. Did either of you experience any particular challenges or difficulties in taking part in the programme? If yes, please can you explain a bit about the challenges or difficulties you faced?
   - Did you find any aspects of the research or therapy sessions difficult or burdensome?
   - If so, what ones?

9. C: How much have you been involved in the programme?
   - Were you present at visits? A few visits or all visits? Which visits?
   - Did you have any involvement in initial discussions about what [the participant] wanted to do within the programme (goal setting)
   - To what extent were you involved in the exercises/activities?
   - How much support did you provide? (What type of support, why?)

Section 3. Exercises and Activities

PHYSIOTHERAPY EXERCISES

The PrAISED programme involves a range of exercises and activities. This includes the physio exercises, other activities within the home (e.g., to help with everyday life, or related to your hobbies/interests), and you may also have been encouraged to go to a group or do some activities (e.g., walking, shopping) outside the home. We are going to talk about each of the different exercises and activities that you have done in the programme and how you found them.

First, I would like to start by just talking about the physio exercises. Please can you tell me a bit about any physio exercises that you have done as part of the PrAISED programme?

1. What exercises do you do? Can you give me some examples?
2. How was it decided which exercises you were to do? (goal setting)
3. How do you feel when doing the exercises? (emotions – enjoyment, fear, boredom)
4. Do you tend to do your exercises at a particular time of day? (what time/why)
5. Do you have a particular place/room (e.g., kitchen, living room) that you do your exercises in? If yes, why? If no, could you find a space with enough space and privacy? How often do you do your exercises? To what extent has this varied? (how/why)
6. Are there any things that made it hard for you to do your exercises regularly? Are there any other things that take up your time?
7. How much time do the exercises take?
8. What do you think influences your motivation to do the exercises?
    - How important is it to you that you do the exercises?
    - How confident do you feel doing the exercises?
    - To what extent do you feel that you can physically do the exercises?
    - To what extent do you feel that you have the information/support that you need in order to know how to do the exercises safely and effectively?
    - How have you found remembering to do the exercises?
    - Have you used any tools/strategies to help you remember to do the exercises? (e.g., planning, reminders, technology etc.)
    - Is there anything else that you feel would help you to do the exercises on a regular basis?
    - To what extent have the views/opinions of others influenced your decision to do the exercises? (carer involvement)
    - Do you think there are any benefits to doing the exercises? If so, what?
9. C: Do you think that [P] has benefited from doing the exercises? If yes, how? If no, why?
    a. Do you think there are any negative effects or risks to doing the exercises? If so, what? [e.g., perceived risk of falls or injury, exacerbating illness]
10. C: Do you think that there are any negative effects or risks to doing the exercises? If so, what?

ACTIVITIES IN THE HOME

Next I would like to find out a bit about any other activities that you did within the home as part of the PrAISED programme. This may be activities that help make it easier for you do the things you want to do on a daily basis or it may be activities related to your hobbies/interests.

Please can you tell me a bit about any other activities within the home that you have done as part of the PrAISED programme? [Occupational therapy activities]

1. What activities did you do?
2. How was it decided which activities you were to do? (goal setting)
3. How did you feel doing the activities? [identify emotions – enjoyment, fear, boredom]
4. Did you tend to do the activities at a particular time of day? (what time/why)
5. How often did you do the activities? Did this vary? (how/why)
6. Are there any things that made it hard for you to do the activities regularly? Are there any other things that take up your time?
7. Did you have a particular place/room (e.g., kitchen, living room) that you did the activities in? If yes, why? If no, could you find a space with enough space and privacy?
8. What do you think influenced your motivation to do the activities?
    - How important were the activities to you?
    - How confident did you feel doing the activities?
    - To what extent did you feel that you could physically do the activities?
    - How much time did the activities take?
    - To what extent did you feel that you have the information/support that you need in order to know how to do the activities safely and effectively?
    - How have you found remembering to do the activities?
    - Have you used any tools/strategies to help you remember to do the activities (e.g., planning, reminders, technology etc.)?
    - Is there anything that you feel would help you to do the activities on a regular basis?
    - To what extent have the views/opinions of others influenced your decision to do the activities? (carer involvement)
    - Do you think there are any benefits to doing the activities? If so, what?
9. C: Do you think that [P] has benefited from doing the activities? If yes, how? If no, why?
    - Do you think there are any negative effects or risks to doing the activities? If so, what [e.g., perceived risk of falls or injury, exacerbating illness]
10. C: Do you think that there are any negative effects or risks to doing the activities? If so, what?

ACTIVITIES AND GROUPS OUTSIDE THE HOME

Next, we are going to talk about any groups or activities that you have done outside of the home as part of the PrAISED project. This may involve attending a group related to a particular hobby or interest of yours. Or it may be other activities undertaken outside the home such as walking, going to the park, or shopping.

Have you got involved in any groups or activities outside the home as part of the PrAISED programme?

If no,

1. Was it something that was suggested to you?
2. How did you feel about getting involved in groups or activities outside the home? [identify emotions – enjoyment, fear, boredom]
   C: How do you feel about [the participant] getting involved in groups or doing activities outside the home?
3. Were there any particular barriers to you getting involved in groups or activities outside?

If yes,

1. Can you tell me a bit about the group/activity? What group did you attend/activity did you do?
2. How was it decided which group/activity you were to do? (goal setting)
3. How did you feel attending the group/doing the activity? [identify emotions – enjoyment, fear, boredom]
4. How often did you attend the group/do the activity? Did this vary? (how/why)
5. Are there any things that made it hard for you to go to the group/do the activity regularly? Are there any other things that take up your time?
6. What do you think influenced your motivation to go to the group/do the activity?
   - How important is the group/activity to you?
   - How confident did you feel attending the group/doing the activity?
   - To what extent did you feel that you could physically do the activity?
   - To what extent did you feel that you have the information/support that you needed to attend the group/do the activity safely?
   - How have you found remembering to go to the group/do the activity?
   - Have you used any tools/strategies to help you remember to go to the group/do the activity (e.g., planning, reminders, technology etc.)?
   - Is there anything that you feel would help you to attend the group/ do the activity on a regular basis?
   - To what extent have the views/opinions of others influenced your decision to attend the group/do the activity? (carer involvement)
7. Do you think there are any benefits to attending the group/doing the activity? If so, what?
8. Do you think there are any negative effects or risks to attending the group/doing the activity? If so, what? [e.g., perceived risk of falls or injury, exacerbating illness]

Section 4. Support and motivation

I'd like to talk a bit now about the support that you received during the PrAISED programme. Within the PrAISED programme you will have been visited by a physiotherapist and an occupational therapist [3-month medium intensity programme] and a support worker [high intensity 12-month programme only].

1. How have you found the support provided by these individuals?
    - Can you tell me a bit about how the clinicians supported you? What was it that they did? How did that make you feel?
2. Was there anything that the clinicians did that influenced your feelings of engagement or willingness to continue with the PrAISED programme?
    - Is there anything that the clinicians have done that you particular liked? If yes, please can you tell me a bit more about that?
    - Is there anything that the clinicians have done which you were not so keen on? If yes, could you expand on that?
3. C: What did you think about the support provided by the clinicians (physiotherapist, occupational therapist and rehabilitation support worker)?
    - Was there anything that you liked about the support provided by clinicians?
    - Was there anything that you disliked about the support provided by clinicians?
4. Is there any other support that you would like to have received? (What/why)

Research

As part of the research you were asked to complete a calendar on a monthly basis noting down any falls and/or exercise undertaken. Please can you tell me a bit about how you found completing the calendar?

1. Did you find the calendar easy or difficult to complete?
2. To what extent did completing the calendar influence your motivation to exercise?
3. How could the calendar be improved?

As part of the PrAISED programme you will have also been involved in other research related activities. At the start of the programme some researchers will have visited you and done some initial cognitive and physical assessments.
4. How did you feel about doing the assessments? (check burden)

If applicable – You were also given a pedometer to measure the number of steps you were doing.
5. How did you feel about having to wear the pedometer? How did wearing the pedometer influence your motivation to exercise?

Section 5. Other exercises/activities outside of PrAISED

1. Please can you tell me a bit about any other exercise, activities or commitments that you do in addition to PrAISED?
2. C: Did participating in the programme have had any impact on your life?
3. How has your involvement in PrAISED influenced your involvement in your other activities?
   - Has being involved in PrAISED interfered with any of your normal daily activities?
4. Do you think there is any link between the amount of exercise you take and your general health and wellbeing?
   - Do you think there are any longer term effects of taking regular exercise?

Section 6. Reflection

We're getting near the end of the interview now, so I would like to check that I have understood your views correctly. So, just to summarise…..

[*Briefly summarise what the participant has said in relation to the following points*]

- Expectations of the programme and whether or not they were met
- Positive aspects of the programme
- Negative aspects of the programme (e.g., risks or harms)
- Particular challenges or difficulties in taking part in the programme (including any burden)
- Influence on willingness to continue participation

1. Are there any ways you think the programme could be improved?
2. To what extent do you think you would be interested in being involved in a similar programme in the future?
3. Is the programme something that you would recommend to a friend?
4. Once the programme has finished, to what extent do you think that you would be interested in continuing with any of the exercises or activities on your own?
5. Do you plan to engage in any other kinds of exercise or activities in the future (what)?

Thank you very much for answering all the questions. Is there anything else you would like to say, or that you feel is important, that we haven't discussed?

Are there any questions you would like to ask me?
